# Acute Administration of n-3 Rich Triglyceride Emulsions Provides Cardioprotection in Murine Models after Ischemia-Reperfusion

**DOI:** 10.1371/journal.pone.0116274

**Published:** 2015-01-05

**Authors:** Hylde Zirpoli, Mariane Abdillahi, Nosirudeen Quadri, Radha Ananthakrishnan, Lingjie Wang, Rosa Rosario, Zhengbin Zhu, Richard J. Deckelbaum, Ravichandran Ramasamy

**Affiliations:** 1 Institute of Human Nutrition, College of Physicians and Surgeons, Columbia University, New York, New York, United States of America; 2 Department of Pediatrics, College of Physicians and Surgeons, Columbia University, New York, New York, United States of America; 3 Department of Medicine, New York University School of Medicine, New York, New York, United States of America; Virginia Commonwealth University Medical Center, UNITED STATES

## Abstract

Dietary n-3 fatty acids (FAs) may reduce cardiovascular disease risk. We questioned whether acute administration of n-3 rich triglyceride (TG) emulsions could preserve cardiac function and decrease injury after ischemia/reperfusion (I/R) insult. We used two different experimental models: in vivo, C57BL/6 mice were exposed to acute occlusion of the left anterior descending coronary artery (LAD), and ex-vivo, C57BL/6 murine hearts were perfused using Langendorff technique (LT). In the LAD model, mice treated with n-3 TG emulsion (1.5g/kg body weight), immediately after ischemia and 1h later during reperfusion, significantly reduced infarct size and maintained cardiac function (p<0.05). In the LT model, administration of n-3 TG emulsion (300mgTG/100ml) during reperfusion significantly improved functional recovery (p<0.05). In both models, lactate dehydrogenase (LDH) levels, as a marker of injury, were significantly reduced by n-3 TG emulsion. To investigate the mechanisms by which n-3 FAs protects hearts from I/R injury, we investigated changes in key pathways linked to cardioprotection. In the ex-vivo model, we showed that n-3 FAs increased phosphorylation of AKT and GSK3β proteins (p<0.05). Acute n-3 TG emulsion treatment also increased Bcl-2 protein level and reduced an autophagy marker, Beclin-1 (p<0.05). Additionally, cardioprotection by n-3 TG emulsion was linked to changes in PPARγ protein expression (p<0.05). Rosiglitazone and p-AKT inhibitor counteracted the positive effect of n-3 TG; GSK3β inhibitor plus n-3 TG significantly inhibited LDH release. We conclude that acute n-3 TG injection during reperfusion provides cardioprotection. This may prove to be a novel acute adjunctive reperfusion therapy after treating patients with myocardial infarction.

## Introduction

Acute myocardial infarction (MI) is a major cause of death despite substantial advancement in diagnosis and therapy in recent decades [1]. Myocardial ischemia/reperfusion (I/R) injury provokes irreversible metabolic and structural changes. Hypoxia, energy depletion, and ion homeostasis alterations characterize ischemic condition, and duration of ischemia has been predictive of severity of myocardial injury [2], [3]. The efficacy of reperfusion, a stage that occurs immediately after ischemia, is also an important factor. If not effective, reperfusion may induce additional, adverse functional and structural tissue damage [4]. A number of basic studies have suggested that strategies to modulate certain pathways of cardiac metabolism during I/R can significantly reduce infarct size. At the molecular level, apoptosis, necrosis, and autophagy have been shown to be involved in myocardial I/R damage [5], [6]. These three different processes likely regulate cell homeostasis and cardiac outcomes after I/R [5], [6].

n-3 fatty acids (FAs) are bioactive nutrients and exert cardioprotective effects in ischemic injury [7], [8], [9]. n-3 FA supplementation can positively affect multiple signaling pathways, such as enrichment of cell membrane phospholipids mainly with eicosapentaenoic acid (EPA) and docosahexaenoic acid (DHA) [10], and modulation of ion channels, receptors and eicosanoids/docosanoids biosynthesis. Furthermore, n-3 FAs are direct ligands for specific transcription factors, affecting inflammatory responses and lipid metabolism [11], [12], [13]. Numerous studies have demonstrated that n-3 FAs possess antioxidant, anti-inflammatory, and anti-apoptotic properties. For instance, n-3 FAs have been shown to be potent activators of AMP-activated protein kinase and histone/protein deacetylase (AMPK/SIRT1) pathway reducing macrophage inflammation and mitochondrial dysfunction [14]. In several ischemic models, n-3 FAs exhibited protective effects by facilitating membrane translocation/activation of AKT and promoting anti-apoptotic and antioxidant pathways [15]. The PI3K/AKT/GSK3β signaling pathway plays a crucial role in inhibition of apoptosis and promoting cell proliferation [16]. Specifically, activation of AKT kinase occurs after ischemic injury and prevents myocardial damage [17].

Current dietary guidelines recommend a daily intake of 1g of EPA + DHA for both primary and secondary prevention of coronary heart disease [18], [19], [20]; although higher pharmacological doses of 3–4 g/day are suggested for hypertriglyceridemia treatment [21], [22]. The GISSI-HF trial found that a low dose of EPA + DHA supplementation significantly reduced mortality compared with placebo in heart failure patients [23]. Apart from these considerations, n-3 FA clinical effects are not yet fully clarified and controversial. The OMEGA trial, for example, did not show benefit of n-3 FA ethyl esters treatment after myocardial infarction [24].

Our approach uses a method of n-3 FA delivery through the administration of lipid emulsions. n-3 triglyceride (TG) emulsions facilitate rapid and sustained increases in n-3 FA delivery to cells [25], [26]. We have previously reported that acute administration of n-3 TG emulsion after ischemic injury is protective in brain [27]. The goals of this study were (a) to investigate whether acute treatment with n-3 TG emulsion during reperfusion time protects the heart from I/R stress, using an *ex-vivo* isolated heart perfusion model and an *in vivo* left anterior descending coronary artery (LAD) occlusion model, and (b) to explore the potential signalling mechanism by which n-3 TG mediate their cardioprotective effects.

## Material and Methods

All studies were performed with the approval of the Institutional Animal Care and Use Committee at Columbia University and New York University School of Medicine, and conform to the *Guide for the Care and Use of Laboratory Animals* published by the US National Institutes of Health (NIH Pub. No. 85–23, 1996). C57BL6 mice (weight between 25–30 g and 12–14 weeks old) were obtained from Jackson Laboratories for our studies. Mice were kept in our animal care facility for a week prior to the studies. All mice were fed a normal chow diet (Teklad Global Diets, Harlan Laboratories). All surgery was performed under anesthesia, and all efforts were made to minimize suffering, as previously described [52].

### Materials

The primary antibodies used were Bcl-2, Beclin-1, PPARγ, p-AKT, total-AKT, p-GSK3β, total- GSK3β (Cell Signaling, USA); and β-actin (BD Biosciences Pharmingen, USA). The secondary antibodies used were anti-rabbit IRdye800, anti-mouse IRdye700 (1:50,000 dilution). SB216763 (3µM), Rosiglitazone (6mg/kg body weight) were purchased from Sigma-Aldrich, USA. Phosphatidylinositol 3-kinase (PI3K)/AKT inhibitor LY-294002 (10 µM) was purchased from Calbiochem, USA. The doses of the inhibitors and agonist used in this study were based on publications in the literature [28]. n-3 fish oil-based emulsion (10 g of TG/100 mL) was commercially prepared intravenous phospholipid-stabilized emulsions, and contained high concentrations of n-3 TG as previously described [27], [29]. n-3 TG emulsions were rich in EPA and DHA (~48% of total FA by weight) [27].

### *In vivo* Left Anterior Descending Coronary Artery (LAD) occlusion

***In vivo* murine model of ischemia-reperfusion injury.** Prior to surgery, mice were anesthetized with isoflurane inhalation (4% induction followed by 1–2.5% maintenance). Subsequent to induction of anaesthesia, mice were orally intubated with polyethylene-60 (PE-60) tubing, connected to a mouse ventilator (MiniVent Type 845, Hugo-Sachs Elektronik) set at a tidal volume of 240 µL and a rate of 110 breaths per minute, and supplemented with oxygen. Body temperature was maintained at 37°C. A median sternotomy was performed, and the proximal left coronary artery (LAD) was visualized and ligated with 7–0 silk suture mounted on a tapered needle (BV-1, Ethicon). After 30min of ischemia, the prolene suture was cut and LAD blood flow restored. Immediately after, intraperitoneal (IP) injection of n-3 TG emulsion (1.5g/kg body weight) was performed and the second injection was done after 60min of reperfusion. Control animals received IP injections of saline solution following the same time course. The chest wall was closed, and mice were treated with buprenorphine and allowed to recover in a temperature-controlled area [30], [31].

### Echocardiogram

*In vivo* transthoracic echocardiography was performed using a Visual Sonics Vevo 2100 ultrasound biomicroscopy system 31. This high-frequency (40 MHz) ultrasound system has an axial resolution of ~30–40 microns and a temporal resolution of >100 Hz. Baseline echocardiography images were obtained prior to myocardial ischemia and post-ischemic images were obtained after 48h of reperfusion. The mice were lightly anesthetized with isoflurane (1.5–2.0 L/min) in 100% O_2_ and *in vivo* transthoracic echocardiography of the left ventricle (LV) using a MS-400 38-MHz microscan transducer was used to obtain high resolution two dimensional mode images. Images were used to measure LV end-diastolic diameter (LVEDD), LV end-systolic diameter (LVESD), LV ejection fraction (EF) and LV fractional shortening (FS) as published earlier [30], [31].

### Infarct size measurement

**Myocardial infarct size determination.** At 48h of reperfusion mice were re-anesthetized, intubated, and ventilated using a mouse ventilator. A catheter (PE-10 tubing) was placed in the common carotid artery to allow for Evans blue dye injection. A median sternotomy was performed and the LAD was re-ligated in the same location as before. Evans blue dye (1.25 ml of a 7.0% solution) was injected via the carotid artery catheter into the heart to delineate the non-ischemic zone from the ischemic zone. The heart was then rapidly excised and sectioned perpendicular to the long axis in 1-mm sections using a tissue chopper. The 1-mm sections was placed in individual wells of a six-well cell culture plate and counterstained with 1% TTC at 37°C to demarcate the nonviable myocardium. Each of the 1 mm thick myocardial slices was imaged and weighed. Images were captured using a Q-Capture digital camera connected to a computer. Images of each slice were captured and then quantified in order to identify myocardial areas: (1) the area representing the non-occluded coronary perfusion area was in blue; (2) the area affected by coronary occlusion and defined as area-at-risk (AAR) for infarction was in red and negative for Evans blue dye; (3) the area within the AAR representing the infarcted zone was white and negative for TTC. We used using computer-assisted planimetry with NIH Image 1.63 software to quantify and distinguish the different areas [30], [31].

### *Ex-vivo* Ischemia and Reperfusion (I/R)

Experiments were carried out as published and modified for use in mice hearts [30], [31]. C57BL6 mice weighting between 25–30 g and 12–14 weeks old were anesthetized by injecting a mixed of ketamine/xylazine [80 mg/kg and 10 mg/kg respectively]. The hearts, rapidly excised, were retrograde perfused through the aorta in a non-recirculating mode, using an isovolumic perfusion system through Langendorff technique (LT), with Krebs-Henseleit buffer, containing (in mM) the following: 118 NaCl, 4.7 KCl, 2.5 CaCl_2_, 1.2 MgCl_2_, 25 NaHCO_3_, 5 glucose, 0.4 palmitate, 0.4 BSA, and 70 mU/l insulin. Perfusion pO_2_ > 600 mmHg was maintained in the oxygenation chamber.

Left ventricular developed pressure (LVDP) was continuously monitored, using a latex balloon placed on the left ventricle and connected to a pressure transducer (Gould Laboratories; Pasadena, CA). Cardiac function measurements were recorded on a 2-channel ADI recorder. LVDP recovery at reperfusion is expressed as (LVDP at reperfusion/ LVDP at baseline) ×100 (%).

The experimental plan included an equilibration baseline period of 30min normoxic perfusion followed by 30min global zero-flow ischemia and then 60min of reperfusion. The flow rate was 2.5 ml/min. The perfusion apparatus was tightly temperature controlled for maintaining heart temperature at 37 ± 0.1°C under all conditions.

The control heart received Krebs-Henseleit buffer; in treated hearts, n-3 TG emulsion was added to the buffer at a concentration 300mg TG/100ml.

Isolated Langendorff-perfused mouse hearts with or without n-3 TG treatment were harvested at 60min of reperfusion and were immediately frozen in liquid nitrogen and stored at -80°C for subsequent molecular analysis.

### Assay of lactate dehydrogenase (LDH)

Myocardial injury was also assessed by measuring the release of lactate dehydrogenase (LDH) from the effluent in the *ex-vivo* I/R system and from blood samples in the *in vivo* LAD system at 48h, using a commercially available enzymatic kit (Pointe Scientific Inc., USA) as published earlier [30], [31].

### Western blot analysis

In isolated Langendorff-perfused mouse hearts, protein concentration was determined using a DC Protein Assay kit (Bio-Rad, USA). Equal amounts of protein were separated by SDS-PAGE (4–12% gradient gels), and proteins were transferred to a nitrocellulose membrane (Invitrogen, USA). After blocking nonspecific binding with the Odyssey blocking buffer (Li-Cor Biosciences, USA), membranes were incubated overnight at 4°C with target primary antibodies (1:1,000 dilution), according to the manufacturer’s instructions. Successively, membranes were incubated with infrared labeled secondary antibodies for 1h at room temperature. The bound complex was visualized using the Odyssey Infrared Imaging System (Li-Cor Biosciences, USA). The images were analyzed using the Odyssey Application Software, version 1.2 (Li-Cor Biosciences, USA) to obtain the integrated intensities.

### Statistical analysis

Data were expressed as the mean ± SD. For assessing the difference between values, the Student’s t test and ANOVA were used. A value of p<0.05 was considered statistically significant.

## Results

### n-3 TG emulsion administration reduces infarct size and maintains cardiac function in *in vivo* LAD model

To test the effectiveness of the acute n-3 TG administration in myocardial ischemic injury, mice were subjected to 30min of ischemia after LAD occlusion. Coronary flow was then restored and myocardial functional recovery during reperfusion was assessed. IP injection of n-3 TG emulsion was performed immediately after ischemia at the onset of reperfusion and again 60min later. To determine whether TG from the n-3 TG emulsions were systemically absorbed by IP injection, our laboratory had previously examined the blood TG levels up to 5hr after the injection [27]. After n-3 TG injection, there was a substantial increase of TG levels up to three fold higher at 1.5hr compared to the baseline, followed by a decrease of levels to baseline at 3 and 5hr [27]. This indicates that n-3 TG had entered into the blood stream and were being catabolized. In comparison, TG levels of saline-injected mice remained constant over the 5hr time period reflecting normal blood TG levels [27].

**Figure 1:**
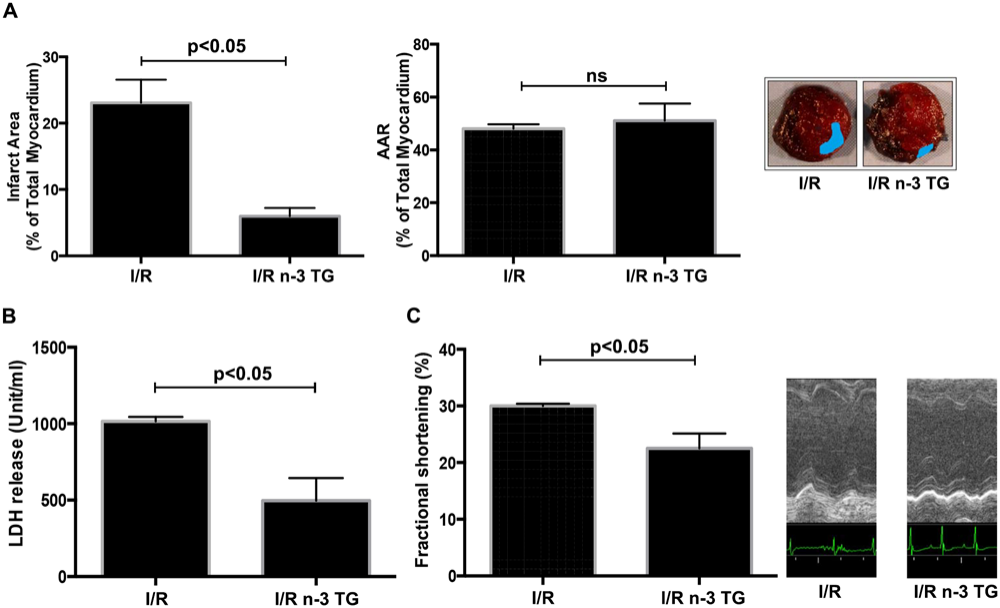
Effects of acute n-3 TG emulsion injection in *in vivo* LAD occlusion model. Mice were subjected to LAD occlusion for 30min followed by reperfusion (48h) with or without acute n-3 TG emulsion injections. Hearts were retrieved at 48h post-LAD ligation and subjected to TTC staining. (A) The analysis of infarct area and area-at-risk (AAR) in the myocardium were determined in I/R vs I/R n-3 TG groups. n = 5–6 mice/group. AAR was no-significant (ns) (B) Plasma collected at 48h was analyzed for total LDH levels in I/R vs I/R n-3 TG groups. n = 5–6 mice/group. (C) Measurements of cardiac function using echocardiography were performed at 48h post-LAD ligation. Changes in % fractional shortening (FS) are reported for each group. n = 5–6 mice/group. Data represent means ± SD.

Mice were then sacrificed at 48h of reperfusion, and sections of heart were stained with TTC to determine the extent of I/R damage comparing n-3 TG treated mice to the control (Fig. 1A). Myocardial infarct size was significantly reduced in n-3 TG emulsion treated mice compared to saline treated mice (p<0.05). AAR (% of total area) was equal in both groups (no-significant, ns) (Fig. 1A). Plasma LDH release, a key marker of myocardial injury, was significantly reduced in n-3 TG treated mice (Fig. 1B). These data indicate that acute treatment of n-3 TG emulsion during reperfusion time markedly reduces injury due to myocardial infarction in mice. Also, echocardiography assessment at 48h of reperfusion showed substantial differences in fractional shortening (FS). We observed a significant recovery of FS in n-3 TG treated mice compared to the control (p<0.05) (Fig. 1C). These data, along with infarct size changes and the attenuation of LDH release during reperfusion, indicate that acute n-3 TG treatment has a cardioprotective effect after I/R injury in *in vivo* murine model and improves viability and function recovery of the heart.

### In *ex-vivo* model, n-3 TG emulsion protects myocardium from I/R injury

**Figure 2:**
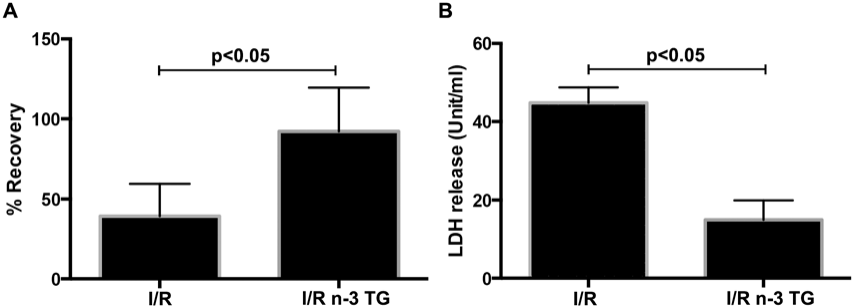
Effect of n-3 TG emulsion *on in vitro* I/R model. (A) Determination of myocardial ischemic injury was assessed by left ventricular developed pressure (LVDP) recovery in hearts subjected to I/R and treated with or without n-3 TG emulsion during reperfusion. (B) Heart perfusates were collected after 1h reperfusion to detect total LDH levels. n = 4–5 mice/group. Data represent means ± SD.

To investigate further the effect of acute intervention with n-3 TG emulsion after ischemic injury, we utilized the *ex-vivo* perfused heart (I/R model). Our experiments showed that administration of n-3 TG emulsion during reperfusion in the *ex-vivo* model significantly improved LVDP recovery after I/R (Fig. 2A), compared to control hearts. Perfusion of the heart with n-3 TG emulsion maintained normal rhythm and LVDP was almost fully restored similar to the pre-ischemic period.

During the reperfusion period, we collected heart perfusates to detect LDH release, as a marker of ischemic injury. LDH release was significantly lower for n-3 TG treated than control hearts, showing that acute n-3 TG treatment exhibits a protective role after ischemic injury (Fig. 2B).

### In *ex-vivo* model, n-3 TG emulsion modulates key signalling pathways linked to I/R injury

**Figure 3:**
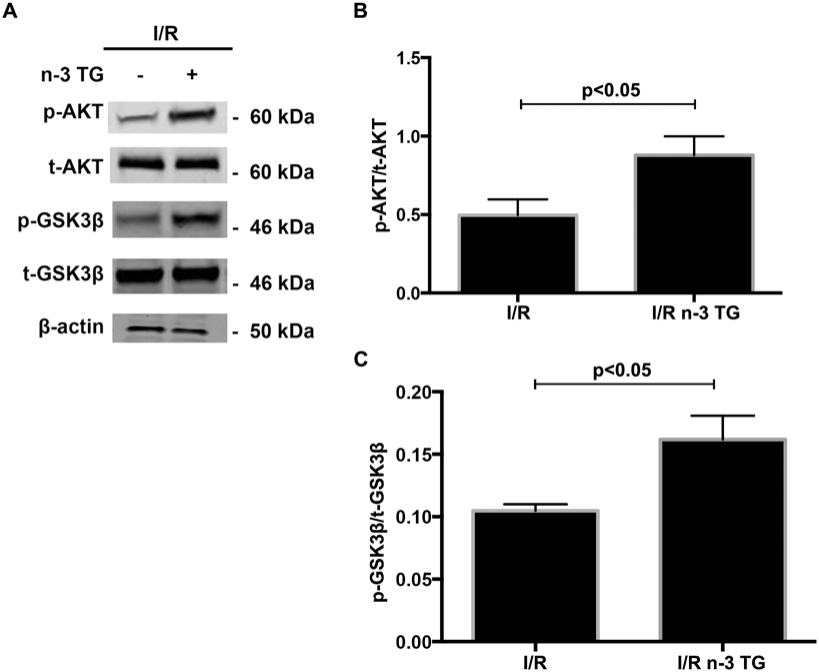
Effects of acute n-3 TG emulsion treatment on signaling pathways in H/R. (A) Western blot analysis of p-AKT and p-GSK3β in hearts subjected to I/R injury with or without n-3 TG emulsion administrated during 1h reperfusion. Bar graphs show (B) p-AKT/t-AKT and (C) p-GSK3β/t-GSK3β protein expression in I/R vs I/R n-3 TG treated hearts. n = 3–4 mice hearts/group. Data represent means ± SD.

**Figure 4:**
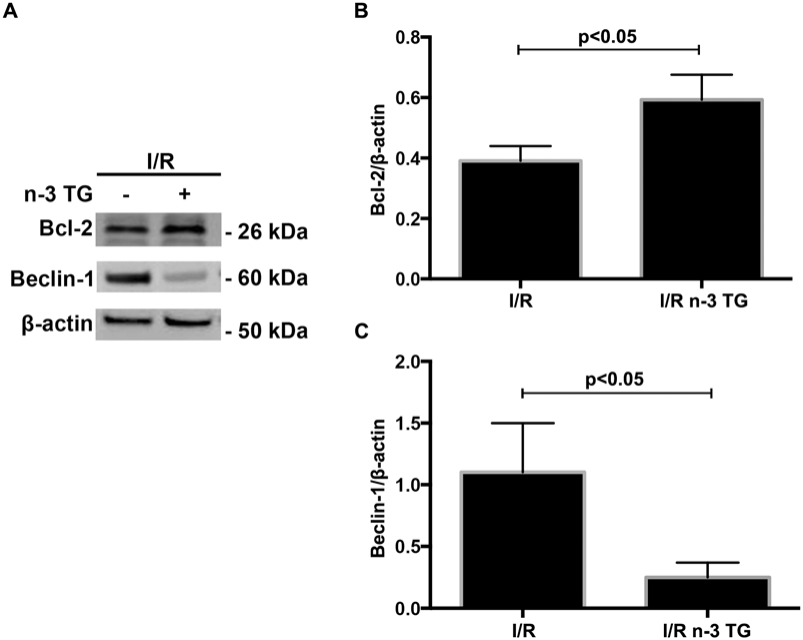
Effects of acute n-3 TG emulsion treatment on apoptosis and autophagy proteins in H/R. (A) Western blot analysis of Bcl-2 and Beclin-1 in hearts subjected to I/R injury with or without n-3 TG emulsion administered during 1h reperfusion. Bar graphics show (B) Bcl-2 and (C) Beclin-1 protein expression in I/R vs n-3 TG treated hearts. n = 3–4 mice hearts/group. Data represent means ± SD

To determine if n-3 TG protects the heart by modulating changes in key signalling pathways linked to I/R injury, we measured p-AKT, p-GSK3β, and Bcl-2 levels in isolated Langendorff-perfused mouse hearts by western blotting analysis. AKT regulates reperfusion injury salvage kinase signalling and members of the Bcl-2 family, which triggers anti-apoptotic pathways. Moreover, AKT regulates the phosphorylation of GSK3β, a pivotal enzyme implicated in mitochondrial regulation. n-3 TG emulsion significantly increased phosphorylation of AKT and GSK3β (Fig. 3) and Bcl-2 protein expression (Fig. 4). Based on these results, n-3 TG reduces apoptosis by activating the PI3K-AKT-GSK3β signalling pathway and anti-apoptotic protein Bcl-2. Bcl-2 interacts with Beclin-1, which is an important protein contributing to autophagosome formation in autophagy [32], [33], As shown in Fig. 4, the expression of Beclin-1 increased after I/R; however n-3 TG treated hearts showed a significant reduction in Beclin-1 protein expression, with associated increase of Bcl-2 protein expression.

**Figure 5:**
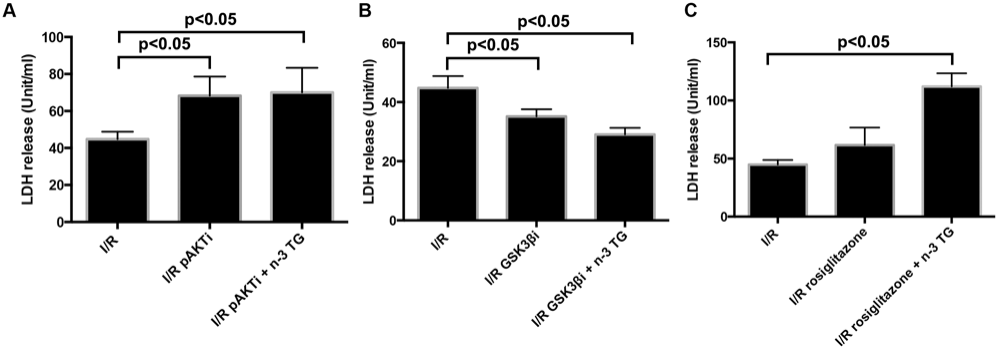
Effects of SB216763, LY-294002, and rosiglitazone treatment on LDH release. Each bar represents LDH release (unit/ml). Hearts were treated with (A) p-AKT inhibitor (10μM LY-294002, p-AKTi), (B) GSK3β inhibitor (3µM SB216763, GSK3βi), and (C) Rosiglitazone, each of them administrated individual or in association with n-3 TG. n = 3–4 mice hearts/group Data represent means ± SD, determined by ANOVA analysis.

To define a potential link between n-3 TG and PI3K/AKT and GSK3β pathways in I/R injury, hearts were treated with the GSK3β inhibitor, SB216763 (3µM), or the phosphatidylinositol 3-kinase (PI3K)/AKT inhibitor, LY-294002 (10μM). Each of these was added to the perfusate at the beginning of the baseline period and this was continued throughout the ischemia and reperfusion period. LDH release was significantly reduced by n-3 TG, however the protection afforded by n-3 TG was abrogated by PI3K/AKT inhibitor, LY-294002 (Fig. 5A). Treatment with SB-216763 plus n-3 TG emulsion significantly inhibited LDH release compared to I/R control hearts (Fig. 5B). These data indicate that AKT and GSK3β are major kinases that contribute to signalling mechanisms implicated in functional and cardiac recovery after I/R injury by acute n-3 TG treatment. Data from LDH release show a major effect by n-3 FA on p-AKT activation, which is upstream to GSK3β.

**Figure 6:**
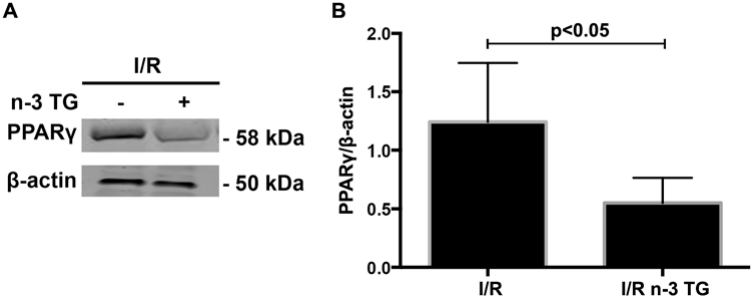
Effects of acute n-3 TG emulsion treatment on PPARγ protein expression. (A) Western blot analysis of PPARγ in hearts subjected to I/R injury with or without n-3 TG emulsion administered during 1h reperfusion. (B) Bar graph shows PPARγ protein expression. n = 3–4 mice hearts/group. Data represent means ± SD.

We previously reported that n-3 TG, in contrast to saturated fatty acids, are able to lower arterial endothelial lipase and macrophage inflammatory markers and these effects are linked to PPARγ [34]. Thus, we hypothesized a potential association of PPARγ in modulating the n-3 TG response after I/R injury. Western blot analysis showed that in n-3 TG treated hearts protein expression of PPARγ was significantly lower compared to control hearts (Fig. 6). To establish the link between PPARγ and n-3 TG cardioprotective effect, we treated the mice with rosiglitazone (6mg/kg body weight, IP injection), a common agonist of PPARγ, 30min before the heart isolation and I/R injury. LDH release was significantly higher in rosiglitazone plus n-3 TG treated hearts vs only rosiglitazone treated hearts (Fig. 5C). These data indicate that PPARγ reduction is linked to cardioprotection afforded by n-3 FAs after I/R injury.

Taken together, these results suggest that PI3K/AKT, GSK3β and PPARγ are important pathways contributing to the cardioprotection afforded by n-3 TG after I/R injury.

## Discussion

n-3 FA supplementation in human studies has shown dissimilar results in the secondary prevention of coronary artery disease [20]. A number of studies in animal models have shown that dietary n-3 FAs provide cardioprotection after I/R, including reduction in myocardial injury [7], [8], [9]. In this study, we demonstrated that the acute administration of n-3 TG emulsion after I/R injury improves cardiac functional recovery, reduces myocardial infarct size, and promotes cell survival through the activation of anti-apoptotic pathways, using two different experimental mouse models.

For our studies, we selected a TG emulsion carrying mainly DHA and EPA, as bioactive molecules, since heart is not able to synthesize sufficiently these essential fatty acids from their precursors. We demonstrated that when administered as a therapeutic approach in post-ischemic treatment protocols, n-3 TG confers cardioprotection. A relevant question is whether this effect seen here is a result of a combined action of DHA and EPA contained in the emulsion or related more to one of these FAs. Previously, our laboratory has addressed this issue in a mouse model of stroke. We have shown that acute post-ischemic treatment with n-3 TG emulsion and pure tridocosahexaenoic acid (Tri-DHA) emulsion decreased cerebral infarct volume while no protection was observed after pure trieicosapentaenoic acid (Tri-EPA) emulsion injection [27]. These observations lead us to investigate the specificity of these FAs in the heart against I/R injury and are the subject of ongoing studies in the laboratory.

In previous studies, our laboratory has also described that it is unlikely that the extra energy supply of n-3 TG emulsions had any protective effects in ischemic model. Indeed, n-3 TG and n-6 TG emulsions injected had similar caloric densities, however n-3 TG provided significant protection but n-6 TG did not [27].

After I/R, cardioprotective mechanisms include the phosphorylation of specific proteins, such as AKT and GSK3β. Our data show that an acute n-3 TG emulsion administration induces cardioprotection through the activation of PI3K/AKT signalling pathways. One of the downstream targets of AKT is GSK3β, which is inactivated by phosphorylation on Ser-9 [35]. Decreased phosphorylation of GSK3β impairs mitochondrial function, as well as functional recovery of the heart. Increases in levels of p-GSK3β, in contrast, correlate with decreased apoptosis. The use of cardioprotective agents has been shown to phosphorylate and inhibit GSK3β action and further protect by inhibiting mitochondrial permeability transition pore (mPTP) opening [36]. Cardiomyocytes from mice with a constitutively active GSK3β were not protected upon treatment with cardioprotective agents, such as erythropoietin as well as exogenous zinc [37], [38]. Other studies have shown a reduction in infarct size and an improvement in post-ischemic cardiac function with the use of GSK3β inhibitors [36], [39], [40]. Consistent with these data of cardioprotection achieved by inhibiting the activity of GSK3β, our results showed significant phosphorylation of GSK3β and a reduction in LDH release in n-3 TG treated hearts.

The timing and the dosage of TG emulsions are of significance as several mechanisms involved in I/R injury arise over a wide time length, from initial minutes to days following ischemic insult, and need to be investigated. For instance, a number of studies demonstrate that Intralipid emulsion administration significantly improves functional recovery of isolated Langendorff-perfused mouse hearts and increased rate pressure [53], [54]. Moreover, post-ischemic intervention with Intralipid emulsion affected the phosphorylation levels of AKT/ERK1/GSK3β [53]. Similarly, our studies in *ex-vivo* model confirmed the involvement of AKT/GSK3β signaling pathway, using however a lower dose for n-3 TG emulsion. Interestingly, administration of 0.3% n-3 TG emulsion rapidly restored heart function, as 50% recovery was observed in LVDP parameter and reduction in LDH release was detected. Also, all hearts were subjected to 30 min of 37°C zero-flow ischemia plus 1h of reperfusion, showing that n-3 TG administration restored functional recovery and is effective even in longer periods of global ischemia.

Li J et al have recently showed that post-ischemic administration of Intralipid, an n-6 FA rich emulsion, protects the heart against I/R injury induced by *in vivo* LAD ligation model [55]. They mainly focused on short-term effects of Intralipid emulsion administered at the onset of 180min reperfusion. Intralipid was as effective as cyclosporine-A in inhibiting the mitochondrial permeability transition pore opening and in decreasing cardiac mitochondrial superoxide production [55]. Of special note, our *in vivo* findings showed that the protective effect of acute n-3 TG administration is prolonged and maintained for a period of 48h after the ischemic insult, as significant reduced extent of I/R damage and improved recovery of FS were detected in n-3 TG treated mice compared to controls.

AKT also regulates various downstream targets including proteins of the Bcl-2 family, linked to mitochondrial apoptotic pathways [41]. Bcl-2-related proteins are known as modulators of anti-apoptotic pathways [42], [43]. Overexpression of the anti-apoptotic protein Bcl-2 has been shown to reduce infarct size [44], [45]. Interestingly, cardiac specific overexpression of Bcl-2 has been shown to reduce the rate of fall in ATP during ischemia and to reduce ischemic acidification [45]. In addition, Bcl-2 has an anti-autophagic role by the inhibition of Beclin-1, contributing to balance cell survival rather than cell death [46]. We detected elevated expression of anti-apoptotic marker Bcl-2 induced by n-3 TG administration after I/R injury, and in contrast, Beclin-1 protein expression was downregulated.

Using the *ex-vivo* model, we selected 60min reperfusion, since it is considered a reliable time to investigate an early apoptotic response and to study the mechanistic trigger of protection [56], [57]. LDH release was significantly lower for n-3 TG treated than control hearts, showing that acute n-3 TG emulsion administration exhibits a protective role after ischemic injury. Along with the attenuation of LDH levels, we detected an increase in Bcl-2 protein expression and a reduction in Beclin-1 protein level, as main markers of apoptosis and autophagy, respectively. These changes observed at 60min of reperfusion may be considered as causative signalling actions rather than consequential. Our findings suggest a need for further studies to determine the involvement of downstream pathways.

PPARγ is a transcription factor mainly involved in lipid metabolism, promoting free FA uptake and TG accumulation [47]. Nevertheless, the exact role of PPARγ regulation in the heart and in the I/R scenario has been under great debate [48]. For example, cardiomyocyte-restricted PPARγ knockout mice develop cardiac hypertrophy, although presenting no changes in lipid metabolism genes [48]. In contrast, murine cardiac PPARγ overexpression leads to dilated cardiomyopathy, accumulation of TG and increased free FA uptake [49]. Our data showed an up-regulation of PPARγ protein expression in the heart after I/R insult; however, this effect was entirely counteracted by the acute administration of n-3 TG emulsion.

Rosiglitazone, a peroxisome proliferator-activated receptor PPARγ agonist, has been reported to have cardioprotective properties during I/R [50]. However, the use of this drug remains controversial as recent meta-analyses and clinical trials have indicated that rosiglitazone therapy is associated with an increased risk of heart failure and myocardial infarction [51]. Activation of PPARγ is involved in lipid accumulation and induces inflammatory pathways [34], [49]. Our finding supports the hypothesis that in I/R injury increased PPARγ expression is “detrimental” and that acute n-3 TG treatment induces a reduction in PPARγ protein levels. Co-administration of a PPARγ agonist and n-3 TG emulsion significantly increases LDH release, abrogating the protective effect of n-3 FAs.

Earlier studies have shown that PPARγ agonists protect hearts by activating AKT and inhibiting JNK signaling pathways [58], [59] However, one study also noted that some actions of PPARγ agonists are independent of its effects on PPARγ [60]. In our studies, n-3 TG emulsion reduced PPARγ activation and was linked to activation of AKT and downregulation GSK3β phosphorylation, thus indicating that in our model AKT-GSK3β axis actions are not linked to PPARγ changes.

Further studies are needed to validate signalling pathways described herein in the *ex-vivo* model and the mechanisms of n-3 TG cardioprotection in the LAD model *in vivo.* Still our results strongly suggested that acute n-3 TG emulsion treatment during reperfusion is cardioprotective. The protective effects of n-3 FAs are trigged by the activation of AKT/GSK3β signalling pathways, promoted by elevations of Bcl-2 expression and Bcl-2/Beclin-1 ratio as well as a downregulation of PPARγ protein levels. Our results indicate the potential therapeutic utility of n-3 TG emulsions as an acute adjunctive therapy for treating patients with myocardial infarction.
