# Correction: Acute Administration of n-3 Rich Triglyceride Emulsions Provides Cardioprotection in Murine Models after Ischemia-Reperfusion

**DOI:** 10.1371/journal.pone.0133546

**Published:** 2015-07-20

**Authors:** The PLOS ONE Staff

Fig 1C appears incorrectly in the published article. Please see the correct Fig 1C here.

**Fig 1:**
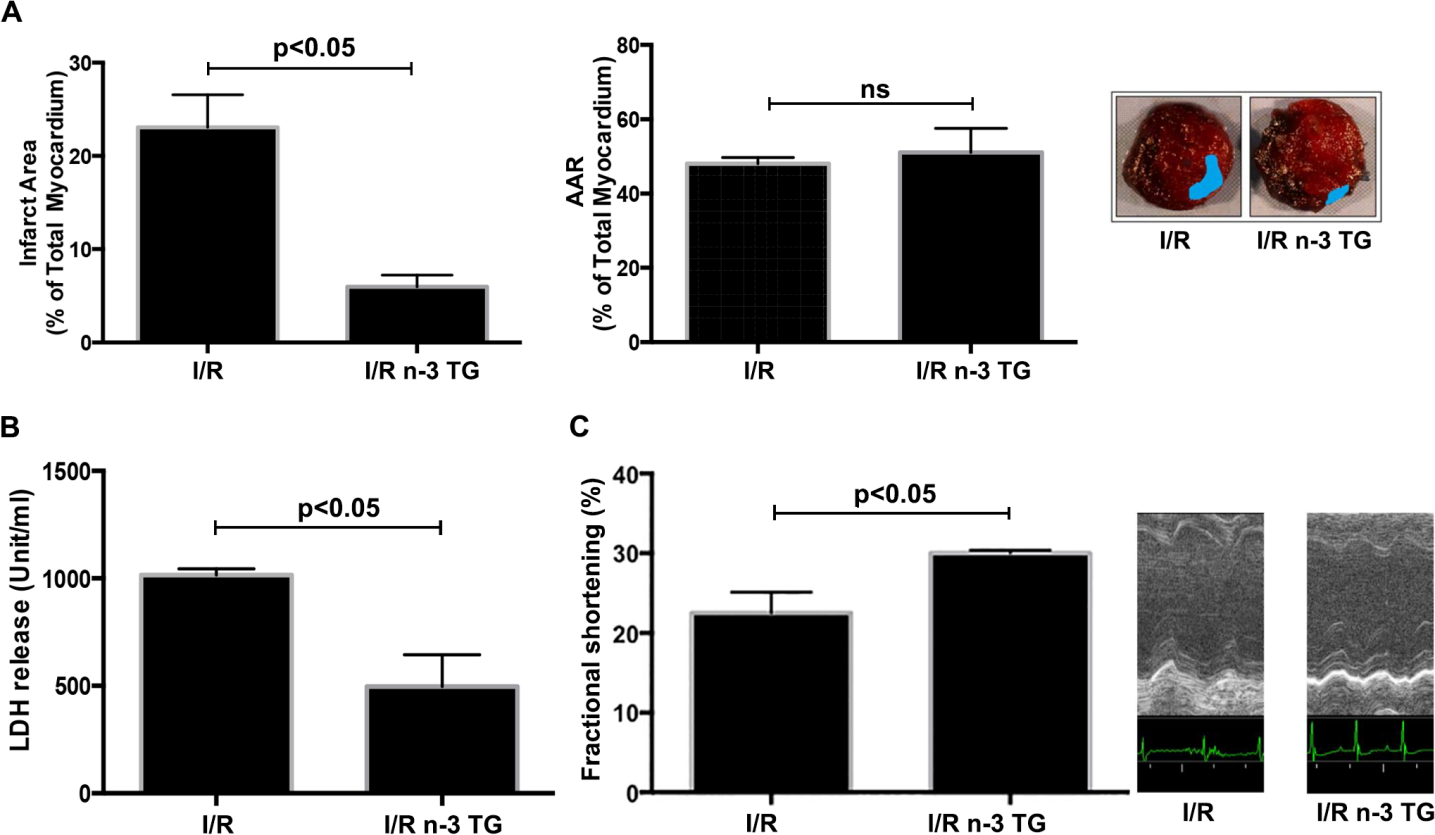
Effects of acute n-3 TG emulsion injection in *in vivo* LAD occlusion model. Mice were subjected to LAD occlusion for 30min followed by reperfusion (48h) with or without acute n-3 TG emulsion injections. Hearts were retrieved at 48h post-LAD ligation and subjected to TTC staining. (A) The analysis of infarct area and area-at-risk (AAR) in the myocardium were determined in I/R vs I/R n-3 TG groups. n = 5–6 mice/group. AAR was no-significant (ns) (B) Plasma collected at 48h was analyzed for total LDH levels in I/R vs I/R n-3 TG groups. n = 5–6 mice/group. (C) Measurements of cardiac function using echocardiography were performed at 48h post-LAD ligation. Changes in % fractional shortening (FS) are reported for each group. n = 5–6 mice/group. Data represent means ± SD.
